# Supplementary material for: OpenMM 7: Rapid development of high performance algorithms for molecular dynamics
Source: PLoS Comput Biol. 2017 Jul 26;13(7):e1005659. doi: 10.1371/journal.pcbi.1005659 (PMC5549999; doi:10.1371/journal.pcbi.1005659)
Supplement: S1 Source Code — (ZIP) [file pcbi.1005659.s002.zip › OpenMM-7.0.1-Source/docs/api-python/generated/simtk.openmm.vec3.Vec3.html]

Vec3 — OpenMM 7.0.1.dev-5e86c4f documentation


# Vec3¶

*class* `simtk.openmm.vec3.``Vec3`¶
:   Vec3 is a 3-element tuple that supports many math operations.

    `__init__`()¶
    :   x.\_\_init\_\_(...) initializes x; see help(type(x)) for signature

    Methods

    |  |  |
    | --- | --- |
    | `count`(...) |  |
    | `index`((value, [start, ...) | Raises ValueError if the value is not present. |

    `__getnewargs__`()¶
    :   Support for pickle protocol 2: http://docs.python.org/2/library/pickle.html#pickling-and-unpickling-normal-class-instances

    `__add__`(*other*)¶
    :   Add two Vec3s.

    `__radd__`(*other*)¶
    :   Add two Vec3s.

    `__sub__`(*other*)¶
    :   Add two Vec3s.

    `__rsub__`(*other*)¶
    :   Add two Vec3s.

    `__mul__`(*other*)¶
    :   Multiply a Vec3 by a constant.

    `__rmul__`(*other*)¶
    :   Multiply a Vec3 by a constant.

    `__div__`(*other*)¶
    :   Divide a Vec3 by a constant.

    `__truediv__`(*other*)¶
    :   Divide a Vec3 by a constant.

    `__contains__`¶
    :   x.\_\_contains\_\_(y) <==> y in x

    `__delattr__`¶
    :   x.\_\_delattr\_\_(‘name’) <==> del x.name

    `__eq__`¶
    :   x.\_\_eq\_\_(y) <==> x==y

    `__format__`()¶
    :   default object formatter

    `__ge__`¶
    :   x.\_\_ge\_\_(y) <==> x>=y

    `__getattribute__`¶
    :   x.\_\_getattribute\_\_(‘name’) <==> x.name

    `__getitem__`¶
    :   x.\_\_getitem\_\_(y) <==> x[y]

    `__getslice__`¶
    :   x.\_\_getslice\_\_(i, j) <==> x[i:j]

        Use of negative indices is not supported.

    `__gt__`¶
    :   x.\_\_gt\_\_(y) <==> x>y

    `__hash__`¶

    `__iter__`¶

    `__le__`¶
    :   x.\_\_le\_\_(y) <==> x<=y

    `__len__`¶

    `__lt__`¶
    :   x.\_\_lt\_\_(y) <==> x<y

    `__ne__`¶
    :   x.\_\_ne\_\_(y) <==> x!=y

    `__reduce__`()¶
    :   helper for pickle

    `__reduce_ex__`()¶
    :   helper for pickle

    `__repr__`¶

    `__setattr__`¶
    :   x.\_\_setattr\_\_(‘name’, value) <==> x.name = value

    `__sizeof__`() → int¶
    :   size of object in memory, in bytes

    `__str__`¶

    `count`(*value*) → integer -- return number of occurrences of value¶

    `index`(*value*[, *start*[, *stop*]]) → integer -- return first index of value.¶
    :   Raises ValueError if the value is not present.

High performance molecular simulation on GPUs


### Navigation

- Application Layer
- Library Layer
  - Core Objects
  - Forces
  - Integrators
  - Extras

©2015, Stanford University and the Authors.
|
Powered by Sphinx 1.3.1
& Alabaster 0.7.7
|
Page source
